# Embryonic Stem Cells Derived from In Vivo or In Vitro-Generated Murine Blastocysts Display Similar Transcriptome and Differentiation Potential

**DOI:** 10.1371/journal.pone.0117422

**Published:** 2015-02-27

**Authors:** Rhodel K. Simbulan, Marlea Di Santo, Xiaowei Liu, Wingka Lin, Annemarie Donjacour, Emin Maltepe, Archana Shenoy, Andrea Borini, Paolo Rinaudo

**Affiliations:** 1 Department of Obstetrics, Gynecology and Reproductive Sciences, University of California San Francisco, San Francisco, California, United States of America; 2 Department of Anatomy, University of California San Francisco, San Francisco, California, United States of America; 3 Department of Pediatrics, University of California San Francisco, San Francisco, California, United States of America; 4 Department of Urology, University of California San Francisco, San Francisco, California, United States of America; 5 Tecnobios Procreazione, Bologna, Italy; Michigan State University, UNITED STATES

## Abstract

The use of assisted reproductive technologies (ART) such as in vitro fertilization (IVF) has resulted in the birth of more than 5 million children. While children conceived by these technologies are generally healthy, there is conflicting evidence suggesting an increase in adult-onset complications like glucose intolerance and high blood pressure in IVF children. Animal models indicate similar potential risks. It remains unclear what molecular mechanisms may be operating during in vitro culture to predispose the embryo to these diseases. One of the limitations faced by investigators is the paucity of the material in the preimplantation embryo to test for molecular analysis. To address this problem, we generated mouse embryonic stem cells (mESC) from blastocysts conceived after natural mating (mESC_FB_) or after IVF, using optimal (KSOM + 5% O_2_; mESC_KAA_) and suboptimal (Whitten’s Medium, + 20% O_2_, mESC_WM_) conditions. All three groups of embryos showed similar behavior during both derivation and differentiation into their respective mESC lines. Unsupervised hierarchical clustering of microarray data showed that blastocyst culture does not affect the transcriptome of derived mESCs. Transcriptomic changes previously observed in the inner cell mass (ICM) of embryos derived in the same conditions were not present in mESCs, regardless of method of conception or culture medium, suggesting that mESC do not fully maintain a memory of the events occurring prior to their derivation. We conclude that the fertilization method or culture media used to generate blastocysts does not affect differentiation potential, morphology and transcriptome of mESCs.

## INTRODUCTION

Thirty-six years after the birth of Louis Brown more than 5 million children have been conceived with the use of ARTs [1]. The procedures are thought to be safe, although a series of obstetrical and perinatal complications have been described following its use [2,3]. Some human studies suggest an increase in long term complications in IVF children [4–6], but others do not [7]. Similarly, several long term health complications such as hypertension, behavioral abnormalities, and glucose intolerance have been described in adult IVF offspring in mice [8–12].

One explanation of how stress during early development may affect long-term health is provided by the developmental origin of health and disease hypothesis (DOHaD) [13]. This theory holds that the embryo or fetus, when exposed to environmental stress, alters its developmental strategy, e.g. gene expression pattern or epigenetic marks, to adapt to the stressful stimulus. The net result is survival but with a predisposition to long term health problems [14]. It is therefore apparent that having a clear understanding of the molecular pathways that are altered at the time of a stressful stimulus could provide important clues about future health of the organism. Analysis of gene expression in IVF and naturally conceived mouse blastocysts has revealed multiple gene expression differences [15–19]. Further, epigenetic differences are thought to be induced by preimplantation embryo culture [20]. However, one of the technical problems faced by investigators is the paucity of tissue available for molecular studies present at the blastocyst stage. The derivation of mouse embryonic stem cells (mESC) from blastocysts would represent a potential solution to this problem. Embryonic stem cells are pluripotent cells that maintain, long-term, the capacity both for self-renewal and differentiation when subjected to the appropriate conditions [21]. The ability of ESCs to divide indefinitely provides an ideal system for the study of early development pathways and offers a potentially unlimited source of cells for complex molecular and epigenetic studies. However, in order to be useful for studies on the mechanism of ART-related changes, embryonic stem cells would need to retain a molecular memory of their original embryo culture environment.

We have recently found that adult mouse offspring generated following transfer of in vivo blastocyst or transfer of in vitro fertilization (IVF) blastocysts cultured in 2 different conditions [optimal conditions (KSOM medium with amino acids IVF_KAA_) or suboptimal conditions (Whitten’s medium)] have different growth patterns and different abilities to handle glucose [22,23]. This suggests that the adult organism maintains a memory of the preimplantation conditions. The aim of this study was to establish an ESC line that faithfully replicated the specific differentiation state of the IVF embryos, and that continually maintained that state. Such lines could be used to study the molecular and epigenetic effects of IVF instead of constantly having to generate a large number of IVF blastocysts. mESC were derived from blastocysts flushed out of the uterus (mESC_FB_, control) or from blastocysts generated by IVF. IVF blastocysts were generated following culture in two different conditions: one thought to be optimal for mouse preimplantation embryo development (mESC_KAA_) and an older medium that is thought to be suboptimal (mESC_WM_). These conditions are widely used in preimplantation embryo culture [20,24]. We compared derivation, proliferation, differentiation and gene expression of the 3 groups of mESC. Our results indicated that IVF does not impact the derivation of mESC and these stem cells do not retain a transcriptomic memory of their preimplantation environment. Further, a comparison of significant genes indicate no overlap in changes seen in the mESC transcriptomes and the transcriptome of the corresponding ICM isolated directly from embryos.

## METHODS

### Animals

All experiments were approved by the Institutional Animal Care and Use Committee of the University of California San Francisco. Animals were provided with nesting material and housed in cages maintained in a constant 12h light/dark cycle at room temperature, with free access to standard chow and tap water. For euthanasia, animals were deeply anesthetized with CO_2_ followed by cervical dislocation.

### *In vitro* fertilization (IVF)

We employed the use of two conditions to assess the influence of culture on the derivation of embryonic stem cells: an optimal culture condition using K^+^ simplex optimized medium with amino acids and 5% O_2_ (IVF_KAA_) and a suboptimal condition using Whitten’s Medium and 20% O_2_ (IVF_WM_). IVF was performed as previously described [19]. Briefly, 6 to 8-week old C57BL/6J female mice were injected with 5 IU pregnant mare serum gonadotropin and with 5 IU human chorionic gonadotropin (hCG) 42–46h later. Oocyte complexes were collected from the ampullae of females 13–15h post-hCG injection and co-incubated with sperm collected from the cauda epididymis of male C57BL/6J mice for 4–6h in the IVF condition described above and washed with fresh media prior to culturing to the blastocyst stage for an additional four days. Control *in vivo* embryos were gathered by mating PMSG and hCG-injected females with males. The presence of a copulation plug the following morning was considered evidence of mating (embryonic day, E0.5). At E3.5, late cavitating blastocysts were flushed out from the uterus of the pregnant female (FB group).

### mESC derivation and culture

mESC derivation was conducted as described, using commercially available reagents [25]. Briefly, E12.5 CF-1 mouse embryonic fibroblasts (MEFs, GlobalStem) were plated on 24-well culture plates the day prior to derivation. Individual late-cavitating blastocysts of similar morphology from each experimental group were transferred onto MEFs in KnockOut DMEM medium (Gibco) supplemented with 15% fetal bovine serum (Hyclone), 1X penicillin/streptomycin, 2mM L-glutamine, 1X non-essential amino acids, 1X β-mercaptoethanol, 1000 U/ml recombinant mouse leukemia inhibitory factor (LIF, Millipore) and 2i, the differentiation inhibitors (30mM CHIR99021 and 10mM PD0325901, Stemgent). The blastocysts were cultured under 20% O_2_ and 5% C0_2_ at 37°C and allowed to attach onto supportive MEFs, hatch and expand without experimental interference for 8 days, though media was changed every two days. After trypsinization, equal cell concentrations were plated onto 12-well culture plates (Corning) and the number of days to reach confluence, the doubling time and percent cell attachment were monitored. The morning after seeding, 100μl of the media was sampled and the number of unattached cells was counted. Derived lines were passaged, via trypsinization, onto a 12-well plate coated with MEFs. Colonies resembling known ESC morphology were detected two days after trypsinization [26]. Cells were allowed to expand and passaged to a 6-well plate coated with MEFs upon development to approximately 70% confluence. Passage number, cell concentration seeded and total cell number upon confluence were recorded. To eliminate the MEFs from mESC culture once the mESC were established, cells were plated onto gelatin-coated 6-well plates for three passages starting from passage 5 [27]. At the eighth passage, plates were examined microscopically to observe whether any feeder cells remained.

### Cell proliferation

To calculate the doubling time, 0.5x10^6^ mESC were plated onto 35mm plates and counted upon reaching approximately 70% confluence. The doubling time of the population was calculated using the equation Y_end_ = Y_start_ x 2^(t/T)^ where T is the doubling time, Y_start_ is the initial concentration of cells, Y_end_ is the final concentration of cells after a period of time, (t). Each line was counted a minimum of three times and the values were averaged [28].

### Sexing of different stem cells

Genomic DNA (gDNA) was extracted from mESC using a QIAamp DNA Mini Kit and performed according to manufacturer’s protocol (Qiagen). 2μl of gDNA was mixed in 1 μl of SRY Primer (F: 5’-TGG GAC TGG TGA CAA TTG TC-3’ R: 5’-GAG TAC AGG TGT GCA GCT CT-3’), 1 μl of IL-3 for internal DNA control (F: 5’- GGG ACT CCA AGC TTC AAT CA-3’ R: 5’-TGG AGG AGG AAG AAA AGC AA-3’), 12.5 μl of 2x PCR Mix (Invitrogen) and 9.5 μl of water for a total of 25 μl reaction. The PCR product was run on a 2% agarose gel mixed with 5 ng of ethidium bromide (Invitrogen) and visualized under UV. The presence of both SRY and IL-3 bands indicated male lines and a single IL-3 band were female lines.

### Affymetrix microarray and analysis

Cells were collected at the eighth passage for microarray analysis. To eliminate the effect of sex as a variable from the analysis, we considered only female lines for this experiment. Total RNA was isolated from three female lines per experimental condition using RNeasy Mini Kit (Qiagen, Valencia, CA) according to manufacturer’s instructions, as previously published [15,17,19]. A total of nine independent biological samples, three per group, were analyzed. RNA concentration was analyzed by spectrophotometry (NanoDrop, Wilmington, DE) and quality was assessed using Agilent Bioanalyzer (Agilent Technologies Inc, Santa Clara, CA). Each sample was diluted to 5 ng/μl and submitted to the Genomic Core Facility of University of California San Francisco for GeneChip hybridization. The samples were hybridized to Affymetrix Mouse Gene 1.0 ST arrays.

To compare the extent of gene overlap between ICM and mESC, we re-analyzed data from a previous microarray experiment, where we analyzed gene differences between ICM derived from in vivo embryo (ICM_vivo_) or from IVF embryos cultured in WM (ICM_WM_) [15]. The ICM were collected from outbred blastocysts (CF-1 x B6D2F1/J) and analyzed using Affymetrix 430 2.0 GeneChip [15]. Because the Affymetrix platforms were different, we could not perform direct comparisons; however, since data analysis was similar, we compared how many genes were in common in the comparisons 1) ICM_FB_ and ICM_WM_ versus 2) mESC_FB_ and mESC_WM_.

### Differentiation of mESC

To study spontaneous differentiation, cells frozen at the seventh passage were thawed, plated in duplicate in 35 mm dishes, and cultured for an additional five passages. LIF was then removed from the culture medium and the medium was changed daily. ESCs were photographed during the thirteenth passage at 72 and 120 hours post-LIF removal and the extent of embryoid body formation was noted. Cells were collected at both time points for the analysis of lineage markers.

### Alkaline phosphatase assay

Alkaline phosphatase (AP) activity of undifferentiated mESC was detected using the Alkaline Phosphatase Staining Kit II (Stemgent), according to manufacturer’s instructions. Lines were examined and photographed under brightfield microscope (S1 Fig.).

### Gene expression analysis

Total RNA was isolated from cells collected in the differentiation study using RNeasy Mini Kit. Reverse transcription was performed through the use of a commercially available first strand cDNA synthesis kit (iScript cDNA, Bio-Rad Laboratories, Hercules, CA) according to manufacturer’s instruction. Real-time quantitative PCR was performed in duplicate using SyBr green PCR supermix (Bio-Rad Laboratories) for markers the following lineage markers: endoderm- *Gata6*, *Pdgfrα*, *Dkk1* [29]; mesoderm- *T*, *Lefty2*, *Meox1* [30]; ectoderm- *Pax6*, *Otx2*, *Fgf5* [31]; pluripotency- *Oct4*, *Sox2*, *Nanog*, *Rex1*, *Klf2* [32] and *β-actin* served as the internal control. Primers were a gift from the laboratory of Dr. Robert Blelloch at University of California, San Francisco. The data were analyzed within the log-linear phase of the amplification curve for each gene, using the comparative threshold cycle method for quantification (Bio-Rad Laboratories). Microarray gene expression data were validated by PCR using 9 genes (S2 Fig.). All the genes tested recapitulated the microarray data.

### Statistics

To determine statistically significant changes in gene expression, analysis of the microarray data was performed using R software version 3.1.0 along with the appropriate Bioconductor (http://www.bioconductor.org/) packages as we have previously described [15]. To remove all possible sources of variation of a non-biological origin between arrays, densitometry values between arrays were normalized using the RMA (robust multiarray) normalization function implemented in the Bioconductor affylmGUI. Statistically significant differences between groups were identified using the rank product non-parametric test implemented in the Bioconductor Rank-Prod package. In fact, applying a Student’s t-test with such a limited number of samples (three in each experimental group) is inappropriate as the obtained statistical significance is not robust and the mean and the standard deviation could be easily biased by outliers. We therefore carried out a non-parametric statistical test as a rough filter to narrow down the list of most relevant genes [33]. This non-parametric method is highly efficient, robust and widely used for microarray data analysis [34]. The Rank Product method has proven to be superior to other statistical methods for microarray data analysis in our and other authors’ experience [35]. Moreover, the rank product approach includes a multiple hypothesis test for raw P-value correction to ascertain a false positive rate similar to false discovery rate correction. Data of the microarray are available at the Gene Expression Omnibus database (http://www.ncbi.nlm.nih.gov/geo).

For Gene ontology (GO) analysis, statistically significant genes were classified into known GO using WEB-based Gene SeT AnaLysis Toolkit (WebGestalt, http://bioinfo.vanderbilt.edu/webgestalt/) to interpret biological functions of genes identified in response to the effect of embryo culture on mESC derivation. Genes represented on the Affymetrix Mouse Gene 1.0ST chip comprise the reference gene list. We restricted the analysis to a significance level of p<0.05 using the hypergeometric statistical method to evaluate the enrichment.

For the genes selected to analyze differentiation, gene expression analysis was performed using Prism5 (Graphpad). Values are presented as mean ± SD. One way ANOVA was used for statistical analysis and Tukey’s post correction was applied to control for multiple comparisons. P<0.05 was considered significant.

## RESULTS

### Mouse embryonic stem cell derivation is not different for IVF or in vivo blastocysts

**Fig 1:**
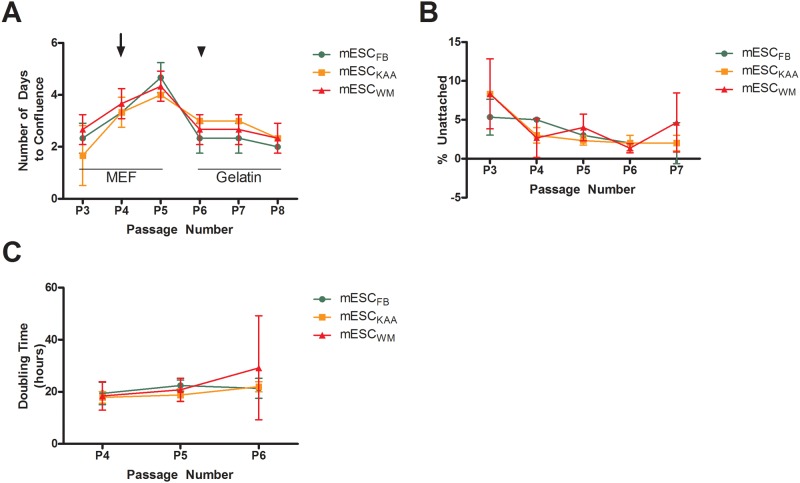
Growth analysis of mESC lines. All mESCs were maintained and seeded with equal cell concentrations on either a MEF feeder layers (Passage 3–5) or gelatin (Passage 6–8). (**A)** Days to confluence. The longer time to confluence in all cell lines on passage 5 (Fig. 1A) was secondary to use larger well size starting from passage 4 (cells were moved from a 24 well plate with a diameter of 15 mm on passage 1 to 3, to a 12 well plate with a diameter of 20 mm on passage 4 and 5 to a well plate with a diameter of 35 mm in passages 6–8). (**B**) Percentage unattached cells after 12h plating. (**C**) Doubling time. Arrow indicates passage to 12 well plates. Arrowhead indicates passage on gelatin.

Blastocysts obtained by flushing the uteri after spontaneous conception (FB) or after *in vitro* culture (IVF_KAA_, IVF_WM_) formed colonies similar in morphology with the same efficiency (Table 1). To assess the impact of embryo culture on the derivation of embryonic stem cells, we compared the growth of mESC after derivation over a period of 8 passages (Fig. 1). The number of days to reach 70% confluence (Fig. 1A), the percentage of cells that did not attach (Fig. 1B) and the doubling time (Fig. 1C) were not different among the groups.

**Table 1:** Total number of cell lines derived from blastocysts.

Condition	Total blastocysts used	Lines Generated	Percentage
**IVF**_**FB**_	62	8	12.90%
**IVF**_**KAA**_	62	10	16.13%
**IVF**_**WM**_	68	6	8.82%

### IVF culture conditions have a subtle influence on the mESC transcriptome

**Fig 2:**
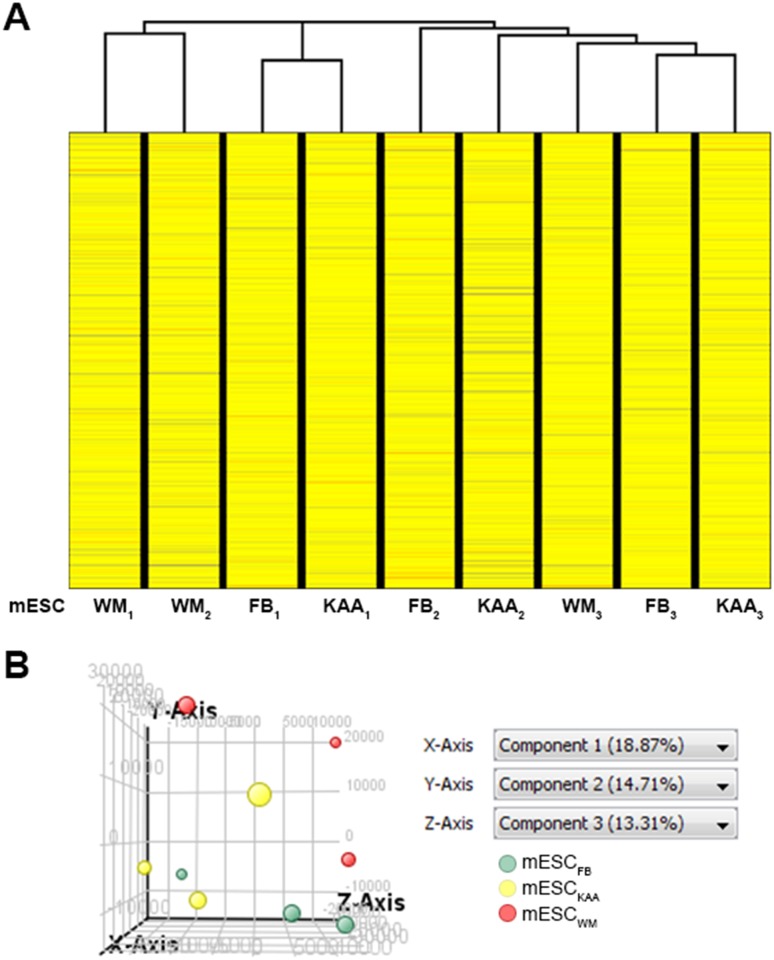
IVF conditions do not influence the transcriptome of the established mESC cell lines. (**A**) Unsupervised hierarchical clustering reveal random clustering of both optimal and suboptimal IVF conditions indicating that IVF has no effect on the global gene expression on the derived mESC lines. (**B**) Principal component analysis of gene expression reveals no distinct clustering between groups.

To ascertain if mESC maintained a memory of their preimplantation conditions, we performed analyses of gene expression utilizing unsupervised hierarchical clustering and principal component analysis (PCA). Hierarchical clustering revealed that culture conditions did not systematically affect the transcriptome, as mESC derived from either IVF conditions did not segregate into distinct branches, instead clustering alongside control mESC_FB_ (Fig. 2A). Further visualization of the expression data by PCA confirms little variation in gene expression profiles of the samples according to the principal component percentages (Fig. 2B).

Overall, only 24 genes were statistically different between mESC_KAA_ and mESC_FB_ and 18 between mESC_WM_ and mESC_FB_ (Table 2). To understand the biological significance of the genes, we carried out a gene ontology pathway analysis. No biological processes were found to be altered between mESC_FB_ and mESC_KAA_ while several pathways involved in metabolic processing, cellular membrane organization and translation initiation were found to be most enriched between mESC_FB_ and mESC_WM_ (Table 3).

**Table 2:** List of statistically significant genes with fold changes from various comparisons.

A. mESC_KAA/FB_		B. mESC_WM/FB_		C. mESC_WM/KAA_	
Gene symbol	Fold change	Gene symbol	Fold change	Gene symbol	Fold change
Copa	1.75	Rpl29	6.75	Rpl29	5.30
Anapc1	1.73	Gbf1	2.42	GM7647	2.94
Tmem234	1.55	GM2964	1.93	GM16088	2.47
Atxn1	1.50	GM17535	1.91	Rpl39L	2.23
VMN2R47	1.47	GM21119	1.83	Zscan4D	2.11
GM5947	1.47	Apol9a	1.52	Mtif2	2.05
VMN2R-PS43	1.46	Gbp2	1.51	GM4340	2.02
Rpl29	1.40	Syngap1	1.5	GM2016	1.86
S100g	1.36	Arfgef1	1.49	GM4014	1.81
Rps2	1.31	Gm10800	1.46	Ythdf1	1.79
Txndc12	1.29	Abhd16a	-1.45	Ube2g1	1.60
Snora21	-1.49	Eif4a2	-1.46	Stxbp4	1.60
GM5634	-1.54	Cox18	-1.47	GM10487	1.53
Hdac5	-1.55	Tcp1	-1.5	Arfgef1	1.47
Tmem129	-1.65	Zkscan3	-1.55	AF067061	1.44
GM20487	-1.66	Mir363	-1.55	Gbf1	1.43
Rnf220	-1.70	Rpl21	-2.05	Cic	1.41
D830030K20RIK	-1.80	Mtif2	-2.33	Dscr3	1.40
GM7647	-1.92			Syngap1	1.36
Hba-A2	-2.07			GM5947	1.32
Pnma5	-2.12			Copa	-1.42
Abhd16a	-2.22			Mir363	-1.51
XLR5D-PS	-2.26			AnapC1	-1.58
Zscan4D	-2.29			Rbm42	-1.60
				Srgn	-1.72
				S100G	-1.78

**Table 3:** Biological processes of statistically significant genes in mESC_WM_/mESC_FB_ comparison.

Pathway	*P* value	GOID	Gene Name
Aromatic, Organonitrogen and Cellular Nitrogen Compound Catabolic process	0.0129	0019439, 0044270, 1901565	Syngap1, Gbp2, Arfgef1
Cellular Membrane Organization	0.0091	0016044	Cox18, Syngap1, Arfgef1
Translation initiation	0.0091	006413	Eif4a2, Mtif2

In previous experiments we had compared the pattern of gene expression between the inner cell mass of IVF and *in vivo*-conceived blastocysts [15] While we could not directly compare the levels of gene expression to those found in mESC, since they were performed on 2 different platforms, we could compare the number and identity of the genes that were different between the two conditions. We hypothesized that if the mESC maintained a high-fidelity memory of their blastocyst environment, the genes that would be altered by IVF in the ICM would be the same genes altered by IVF in the mESC. While 329 genes were different between ICM_WM_ and ICM_FB_ [15] only 18 genes were different between mESC_WM_ and mESC_FB_ and none of the genes overlapped.

### mESC from different conditions are similar in differentiation potential

**Fig 3:**
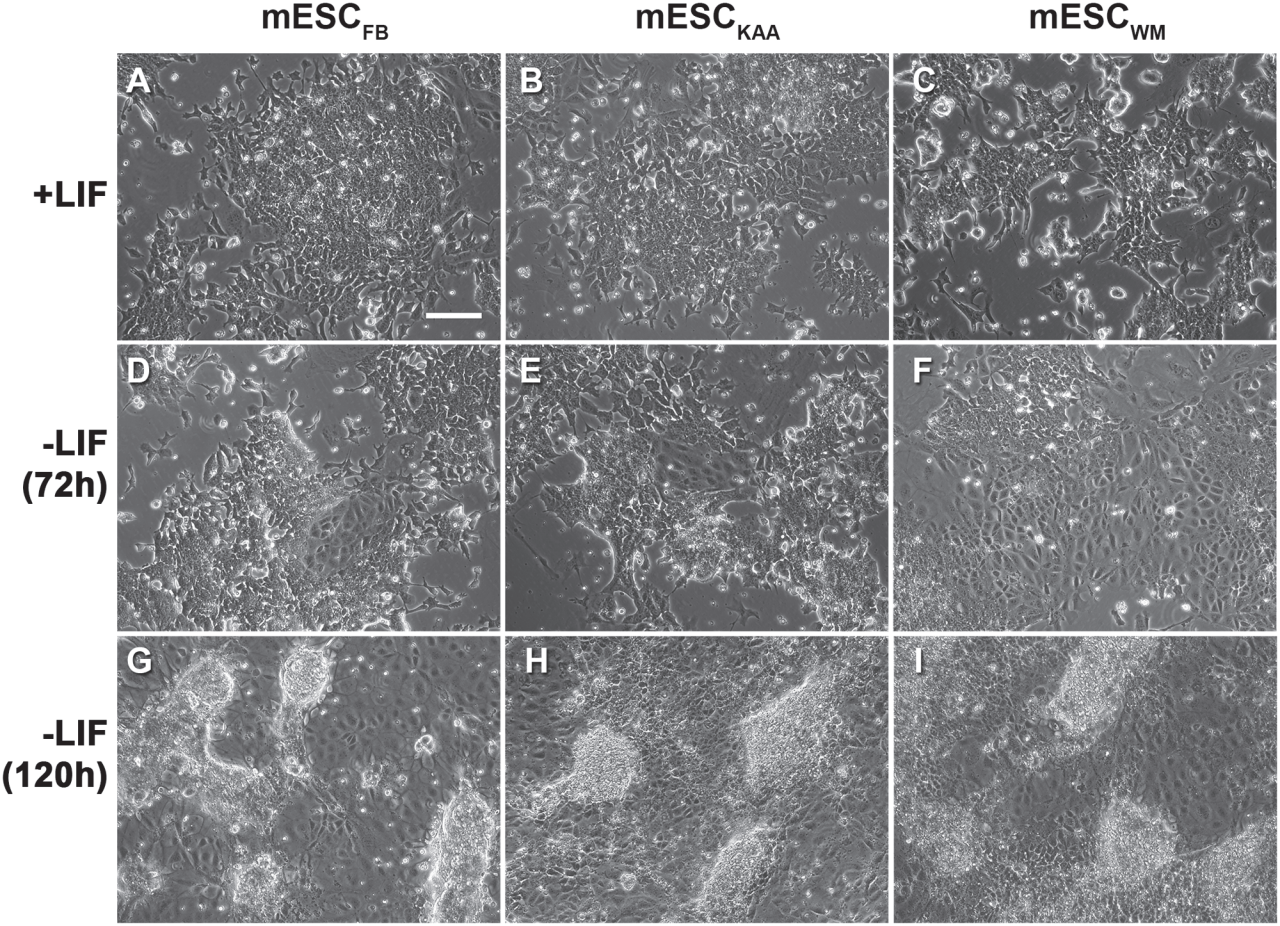
Morphology of mESC. (**A-C**) Undifferentiated stem cells. (**D-F**) 72h after LIF removal. (**G-I**) 120h after LIF removal. Embryoid bodies are clearly visible at this stage. All photographs were taken under a 10X objective and the scale bar represents 20μm. No differences in morphology between conditions were observed at any time points.

**Fig 4:**
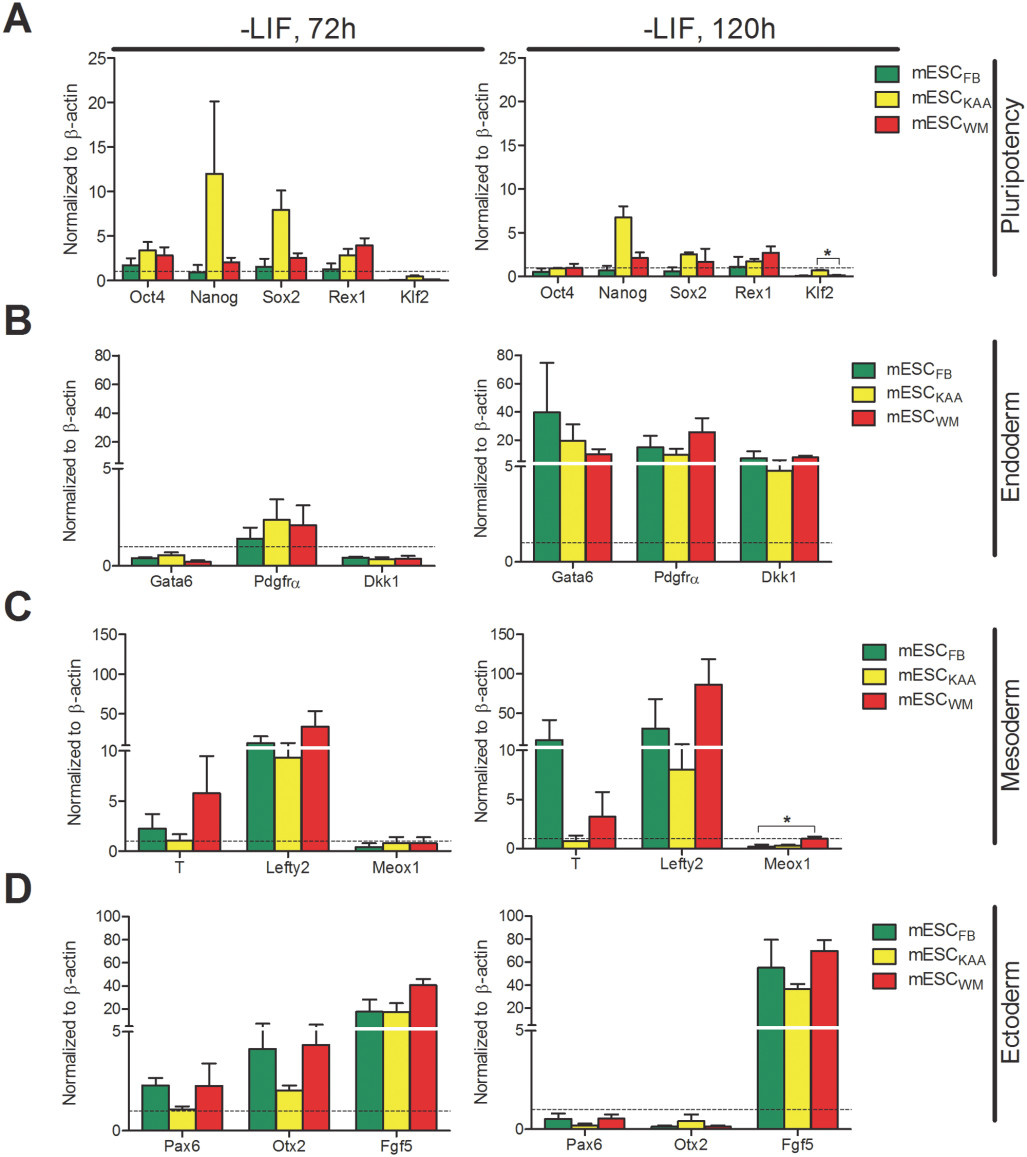
Expression of lineage markers by mESC during spontaneous differentiation. Fold changes of each gene compared to their expression at time zero (horizontal line at 1) (**A**) Pluripotency markers 72 and 120h after the removal of LIF. (**B**) Ectoderm markers 72 and 120h after the removal of LIF. (**C**) Mesoderm markers 72 and 120h after the removal of LIF. (**D**) Endoderm markers 72 and 120h after the removal of LIF. *p<0.05, one-way ANOVA with Tukey’s post-hoc correction.

We next evaluated whether the method of conception influenced a stem cell’s tendency to differentiate into a particular lineage. Pluripotent mESC from the 3 groups, grown in the presence of LIF, appeared to be morphologically similar (Fig. 3A-C). No differences among the three stem cell groups were observed after the removal of LIF for 72h (Fig. 3D-F) or 120h (Fig. 3G-I). After 120h, distinct colonies resembling embryoid bodies, alongside a layer of cells with a morphology characteristic of epithelial cells, were observed in all three groups of stem cells. We also analyzed differentiation by comparing the expression of genes known to be markers of selected lineages. In general the cells showed similar, decreased expression of pluripotency markers (Oct4, Nanog, Sox2, Rex1 and Klf2) 72h after spontaneous differentiation by LIF removal. The only statistically significant difference was between MESC_WM_ and mESC_KAA_ Klf2 levels at 120h (Fig. 4A). No statistical differences were found in the expression of markers of endoderm (Gata6, Pdgfrα, Dkk1) and ectoderm (Pax6, Otx2, Fgf5) (Fig. 4B and D). Among the markers of mesoderm (T/Brachyury, Lefty2 and Meox1) the only statistically significant difference was between mESC_WM_ and mESC_FB_ in Meox1 levels at 120h after LIF removal (Fig. 4C).

## DISCUSSION

Embryos growing in culture adapt to their suboptimal environment with various short-term changes that have long-term implications. Documenting the molecular changes that accompany this adaptation will help to uncover the mechanism of reprogramming. This has wide clinical relevance, since preimplantation embryo culture is widely used in clinical practice to treat patients with infertility [36]. Animal studies suggest that the manipulation of gametes and embryos during the preimplantation phase alone can result in the alteration of adult metabolic health [8,10], cardiovascular dysfunction [12] and changes in behavior [24,37]. Further it appears that even the composition of the medium or the oxygen concentration used to culture embryos might directly contribute to perturbations in gene expression, including the expression of imprinted genes [9,19]. For example the imprinted *H19* gene exhibits biallelic expression after embryo culture in WM, but not after culture in KSOM medium with amino acids. [20]. Overall culture in WM is known to be stressful to embryos and culture under this conditions results in a more altered pattern of gene expression changes and impaired development [17]. Fetuses generated from embryos grown in WM show abnormalities of fetal and placenta growth [38] and offspring generated by IVF in WM show a clear glucose-intolerant phenotype [8,10]. The composition of WM is quite different from KSOM as it has higher concentrations of glucose (5.5mM vs 0.2mM) and is not supplemented with amino acids or glutamine [39,40]. It is important to know the mechanism by which these suboptimal conditions affect long-term health.

The present study was designed to examine whether mESC derived from blastocysts generated in vivo or in vitro in different conditions (WM or KAA) maintain a memory of their perturbed early environment. Molecular studies of preimplantation embryos are hindered by the limited amount of material available. Derivation of embryonic stem cells would provide an excellent solution to the problem by providing abundant material to test.

The major conclusion of these studies is that mESC examined at a realtively early passage (passage 8 in our case) are not a suitable source of cells for studying the mechanism of embryonic reprogramming by IVF. Several lines of evidence lead to this conclusion. First, mESC derived from embryos cultured in optimal (mESC_KAA_) and suboptimal conditions (mESC_WM_) are morphologically and transcriptomically similar to mESC derived from control embryos (mESC_FB_). In fact, hierarchical clustering indicates that mESCs derived from optimal IVF conditions share a similar transcriptomic signature as those derived from *in vivo* embryos. On the contrary, unsupervised clustering of gene expression of blastocysts cultured in KSOM with amino acids or in vivo showed separate clustering [17]. In addition, the number of genes found to be significantly altered in mESC are much fewer compared to ICM from our previous study with no identical biological processes observed between mESC and ICM. Given these different results in blastocysts and mESC, we conclude that mESC cannot be used for molecular studies to dissect out the effect of the method of conception or culture on gene expression or epigenetic changes. It is still possible that mESC generated at earlier passages (2–3, for example) might maintain a closer memory of the blastocyst derivation methods. Future studies should test this possibility.

Secondly, the fact that there was no overlap among the gene changes between ICM and mESC, indicate that the derivation process and repeated passaging needed to generate stem cells induces multiple changes in the transcriptome of mESC so that these cell lines have only minimal resemblance to the transcriptome of ICM. Similar results were found in a study comparing the transcriptome of human ES lines with isolated human ICM cells [41]. Comparison of global gene expression between individual ICM clusters and human embryonic stem cells indicated that these two cell types are significantly different in regards to gene expression, with fewer than one half of all genes expressed in both cell types [41]. Of note, Horii et al. reported that epigenetic differences between ICM and ESC found at passage 2 were eventually lost by passage 5 [42]. Given the results showing transcriptome similarity, we did not examine the methylation status of genes in this study. Prior work has examined DNA methylation differences in mESC generated in vivo or in vitro at selected loci [42,43]. These authors found epigenetic differences between mESC originated from in vivo or in vitro blastocysts only after the initial passages, while the differences disappeared at later passages, suggesting an effect of extended culture on mESC epigenome [42]. More specifically the authors found modifications of the methyltransferase Dnmt3b in *in vitro* cultured blastocysts when compared to in vivo blastocysts. These differences persisted in mESC derived from in vitro generated blastocysts [43]. If present, epigenetic changes in our system do not appear to result in alterations of significant gene expression.

One caveat for this study is that we performed microarray analysis only on female stem cell lines while the original blastocysts experiments were performed both in males and female lines. We decided to analyze only female mESC lines to minimize the effect of sex on gene expression. In fact, up to one-third of transcripts are differentially expressed in male and female blastocysts, with particular variation in glucose and protein metabolic pathways [44]. Instead, the blastocyst gene expression data [17] were obtained before the publication of the above mentioned sexual dimorphic gene expression data. One additional limitation is that we only studied stem cell differentiation potential by embryoid body formation and a more robust conclusion regarding the cell’s differentiation potential could be made using additional differentiation conditions. While a single differentiation strategy is accepted [42], it is possible that other differentiation methods (culturing in vitro on Matrigel, BMP4 or in vivo with teratoma formation) would have obtained different results. However, the primary goal of our investigation was to test if undifferentiated stem cells are a good model to investigate ICM of embryos.

In summary, we found that the original blastocyst culture condition from which mESC lines are derived from result in cell lines with similar transcriptomic signatures, similar morphology and differentiation potential. Most importantly, the gene expression differences between ICM and mESC are of such magnitude as to question the validity of using mESC to dissect out the effect of the method of conception or the culture media used to culture embryos. Overall, we provide evidence that mESC are not suitable substitutes for mouse blastocyst molecular and epigenetic studies.

## Supporting Information

S1 Fig Alkaline phosphatase staining of undifferentiated mESC. Alkaline phosphatase activity of undifferentiated mESC lines (mESC_FB_, mESC_KAA_ and mESC_WM_). All photographs were taken under a 10X objective and the scale bar represents 20μm.
(TIF)

S2 Fig Validation of microarray data by qPCR of selected genes. qPCR of lineage markers measured on undifferentiated mESC recapitulate the result of the microarray platform.
(TIF)
