# Supplementary figures and images for: Embryonic Stem Cells Derived from In Vivo or In Vitro-Generated Murine Blastocysts Display Similar Transcriptome and Differentiation Potential
Source: PLoS One. 2015 Feb 27;10(2):e0117422. doi: 10.1371/journal.pone.0117422 (PMC4344309; doi:10.1371/journal.pone.0117422)

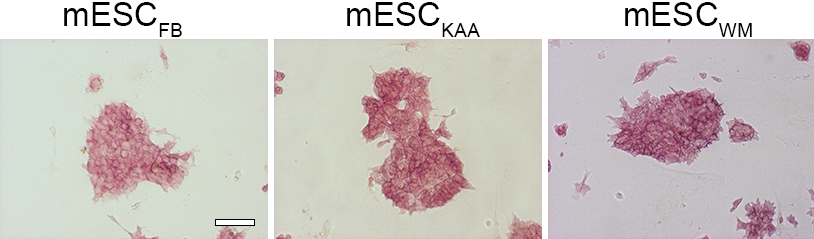

Supplement: S1 Fig — Alkaline phosphatase activity of undifferentiated mESC lines (mESCFB, mESCKAA and mESCWM). All photographs were taken under a 10X objective and the scale bar represents 20μm. (TIF) [file pone.0117422.s001.tif]

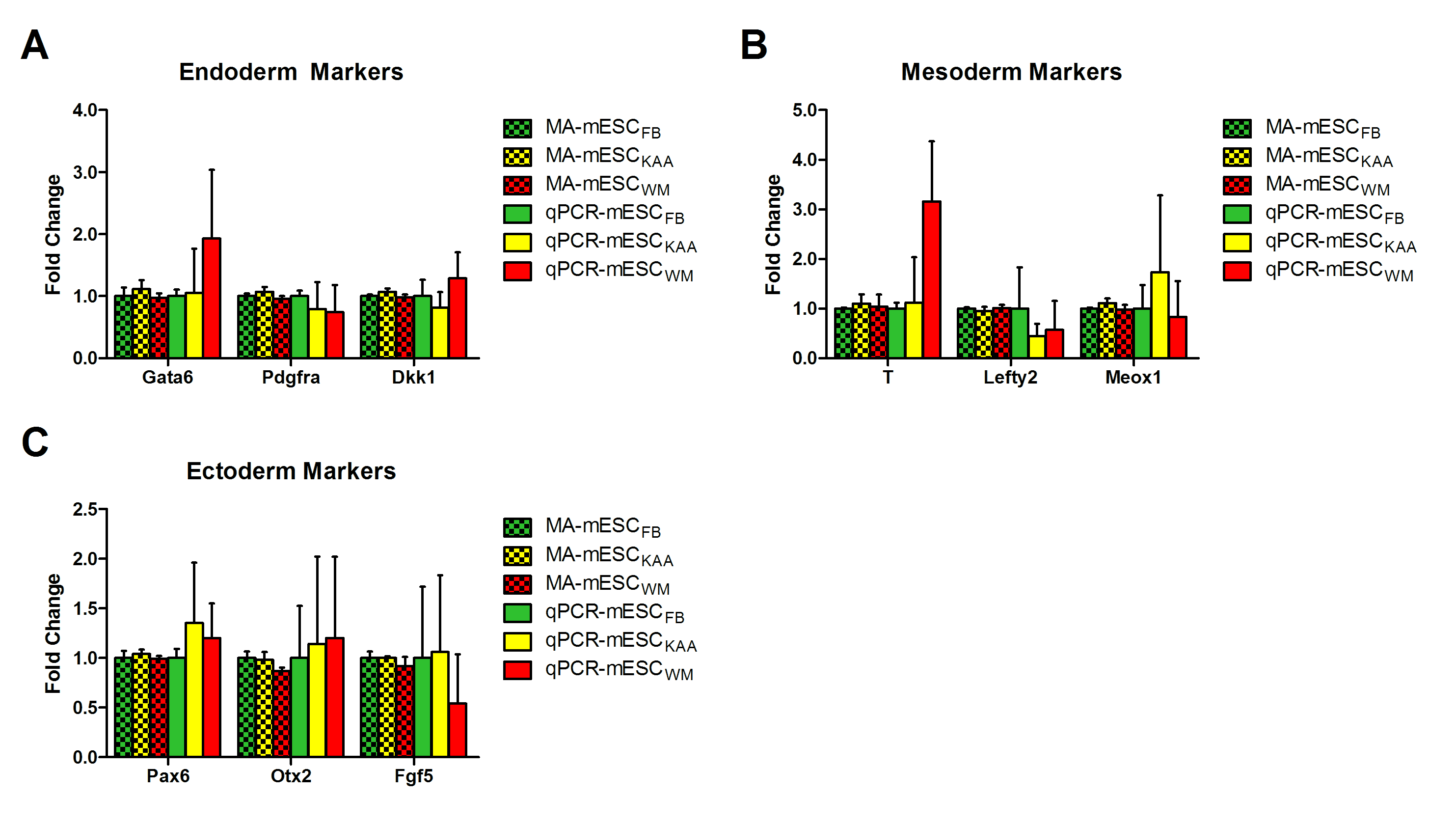

Supplement: S2 Fig — qPCR of lineage markers measured on undifferentiated mESC recapitulate the result of the microarray platform. (TIF) [file pone.0117422.s002.tif]
